# The causal relationship between anti-diabetic drugs and gastrointestinal disorders: a drug-targeted mendelian randomization study

**DOI:** 10.1186/s13098-024-01359-z

**Published:** 2024-06-26

**Authors:** Mingyan Ju, Tingting Deng, Xuemin Jia, Menglin Gong, Yuying Li, Fanjie Liu, Ying Yin

**Affiliations:** 1 College of Acupuncture and moxibustion, Shandong University of Traditional Chinese Medicine, Jinan, China; 2 College of Traditional Chinese Medicine, Shandong University of Traditional Chinese Medicine, Jinan, China; 3 Shandong First Medical University & Shandong Academy of Medical Sciences, Jinan, Shandong China; 4 Bone Biomechanics Engineering Laboratory of Shandong Province, Shandong Medicinal Biotechnology Center (School of Biomedical Sciences), Neck-Shoulder and Lumbocrural Pain Hospital of Shandong First Medical University, Shandong First Medical University & Shandong Academy of Medical Sciences, Jinan, China; 5 Affiliated Hospital of Shandong University of Traditional Chinese Medicine, Jinan, China

**Keywords:** Antidiabetic drugs, Diabetes, Gastrointestinal diseases, Drug targets, Mendelian randomization study

## Abstract

**Background:**

The incidence of diabetic gastrointestinal diseases is increasing year by year. This study aimed to investigate the causal relationship between antidiabetic medications and gastrointestinal disorders, with the goal of reducing the incidence of diabetes-related gastrointestinal diseases and exploring the potential repurposing of antidiabetic drugs.

**Methods:**

We employed a two-sample Mendelian randomization (TSMR) design to investigate the causal association between antidiabetic medications and gastrointestinal disorders, including gastroesophageal reflux disease (GERD), gastric ulcer (GU), chronic gastritis, acute gastritis, Helicobacter pylori infection, gastric cancer (GC), functional dyspepsia (FD), irritable bowel syndrome (IBS), ulcerative colitis (UC), Crohn’s disease (CD), diverticulosis, and colorectal cancer (CRC). To identify potential inhibitors of antidiabetic drug targets, we collected single-nucleotide polymorphisms (SNPs) associated with metformin, GLP-1 receptor agonists, SGLT2 inhibitors, DPP-4 inhibitors, insulin, and its analogs, thiazolidinediones, sulfonylureas, and alpha-glucosidase inhibitors from published genome-wide association study statistics. We then conducted a drug-target Mendelian randomization (MR) analysis using inverse variance weighting (IVW) as the primary analytical method to assess the impact of these inhibitors on gastrointestinal disorders. Additionally, diabetes was selected as a positive control.

**Results:**

Sulfonylureas were found to significantly reduce the risk of CD (IVW: OR [95% CI] = 0.986 [0.978, 0.995], *p* = 1.99 × 10^− 3^), GERD (IVW: OR [95% CI] = 0.649 [0.452, 0.932], *p* = 1.90 × 10^− 2^), and chronic gastritis (IVW: OR [95% CI] = 0.991 [0.982, 0.999], *p* = 4.50 × 10^− 2^). However, they were associated with an increased risk of GU development (IVW: OR [95%CI] = 2 0.761 [1.259, 6.057], *p* = 1 0.12 × 10^− 2^).

**Conclusions:**

The results indicated that sulfonylureas had a positive effect on the prevention of CD, GERD, and chronic gastritis but a negative effect on the development of gastric ulcers. However, our research found no causal evidence for the impact of metformin, GLP-1 agonists, SGLT2 inhibitors, DPP 4 inhibitors, insulin and its analogs, thiazolidinediones, or alpha-glucosidase inhibitors on gastrointestinal diseases.

**Graphical abstract:**

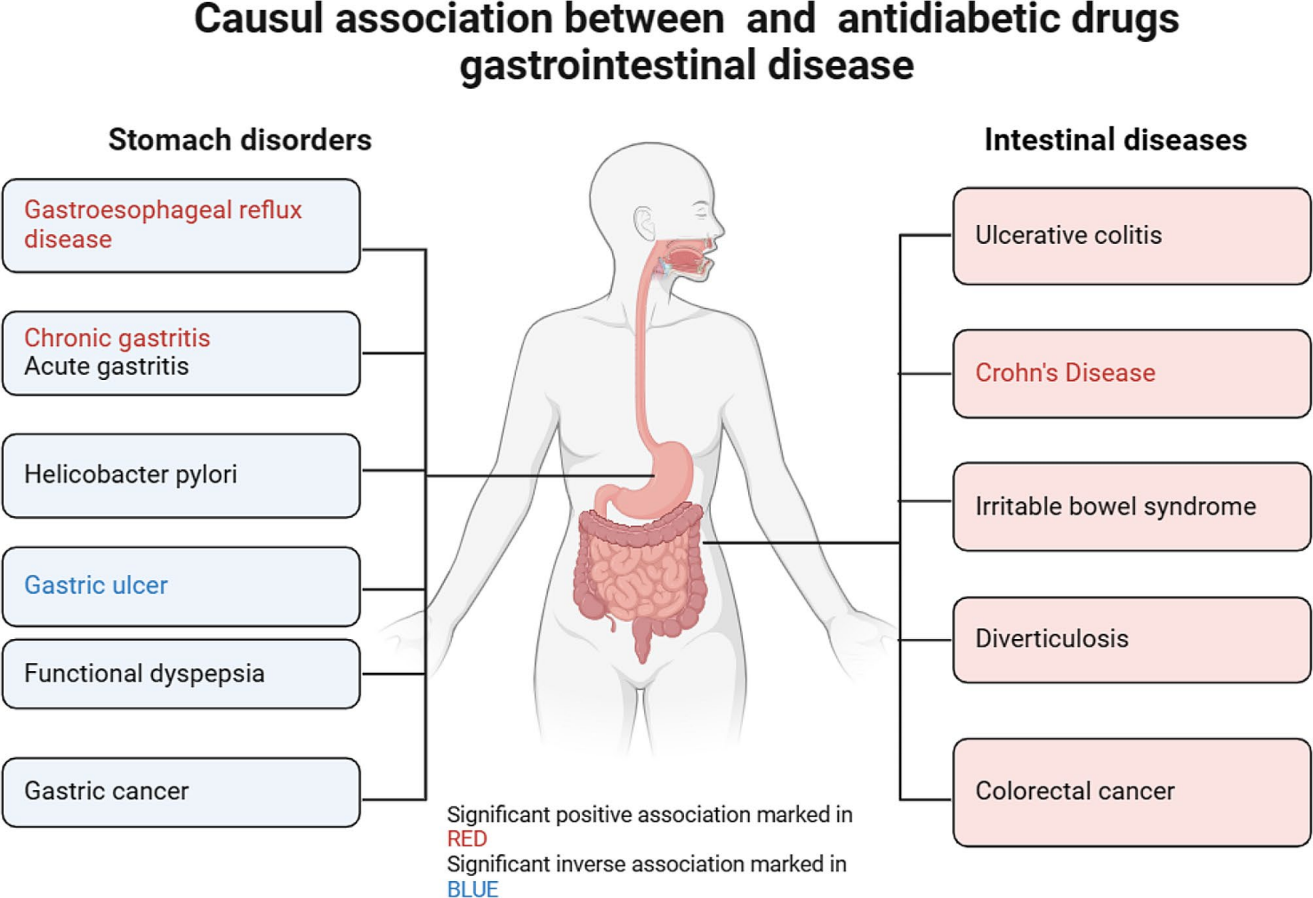

**Supplementary Information:**

The online version contains supplementary material available at 10.1186/s13098-024-01359-z.

## Background

According to the United European Gastroenterology, the incidence of gastrointestinal diseases is increasing each year [1]. Studies on the demography of aging in the elderly and the epidemiology of gastrointestinal diseases show a strong association between age and a higher prevalence of these disorders. As the population ages, the prevalence of gastrointestinal diseases is expected to rise [2]. Many factors contribute to the development of gastrointestinal disorders, and research by Babu Krishnan et al. indicates that diabetes can lead to a variety of gastrointestinal complications [3]. A prospective study demonstrated a higher prevalence of GERD symptoms among patients with type 2 diabetes compared to the general population [4]. Additionally, a systematic review of meta-analyses found that diabetes significantly increases the risk of inflammatory bowel disease [5]. Retrospective studies have also shown a correlation between autoimmune gastritis and type 1 diabetes [6]. Furthermore, patients with diabetes are more susceptible to Helicobacter pylori infection [7, 8]. A systematic review and meta-analysis of cohort studies conducted by Jinru Guo et al. demonstrated that the risk of gastric cancer is higher in individuals with diabetes, with the risk varying based on the duration since the onset of diabetes [9]. Chin-Hsiao Tseng’s population-based analysis in Taiwan similarly suggests that people with diabetes have a higher risk of developing stomach cancer [10, 11]. Moreover, patients with diabetes have a significantly higher risk of death from colon cancer [12]. A Mendelian Randomization (MR) study suggests that type 2 diabetes and impaired glycemic homeostasis raise the risk of gastrointestinal diseases [13]. Antidiabetic drugs may also be linked to gastrointestinal diseases. Animal experiments by Isabela R.S.G Noleto et al. showed that metformin has a protective effect on the gastric mucosa and prevents peptic ulcer formation in hyperglycemic rats [14]. Furthermore, metformin possesses anti-inflammatory properties and is used to treat inflammatory bowel disease [15]. Metformin may reduce the risk of inflammatory bowel disease in people with type 2 diabetes [16]. It also reduces the risk of inflammatory bowel diverticulosis in patients with type 2 diabetes [17]. Retrospective cohort studies have found that metformin reduces the risk of gastric and colorectal cancer in patients with diabetes [18–20]. Additionally, studies have shown that metformin reduces the risk of Helicobacter Pylori (HP) infection [21], and insulin use is significantly associated with a higher incidence of HP eradication [22]. Audrius Dulskas et al. found an increased risk of stomach cancer in patients with diabetes treated with sulfonylureas [23]. However, a retrospective population-based cohort study conducted on the Italian population revealed a reduction in GC risk associated with sulfonylurea usage [24].

Observational studies on the relationship between different antidiabetic drugs and gastrointestinal disorders have produced controversial results. No studies have yet explored the causal relationship between antidiabetic drugs and gastrointestinal disorders. MR is an analytical method used to study causal relationships between exposures and clinically relevant outcomes [25]. It can predict adverse drug reactions and provide opportunities for drug repurposing [26]. MR of drug targets can reflect the effects of drug use by using genetic instrumentation within or near the target gene to analyze genetic variants that mimic the pharmacological inhibition of drug targets [27].

In this study, we used SNPs in or near target as pharmacogenetic proxies to explore the causal relationship between antidiabetic drugs and gastrointestinal diseases including GERD, GU, chronic gastritis, acute gastritis, Helicobacter pylori infection, gastric cancer, FD, IBS, UC, CD, diverticulosis, and CRC. The results can guide medication choices for patients with diabetes with gastrointestinal complications and suggest potential gastrointestinal prevention strategies for future clinical trials. This can improve the happiness index and quality of life for patients with diabetes, especially elderly people.

## Methods

### Research design

**Fig. 1:**
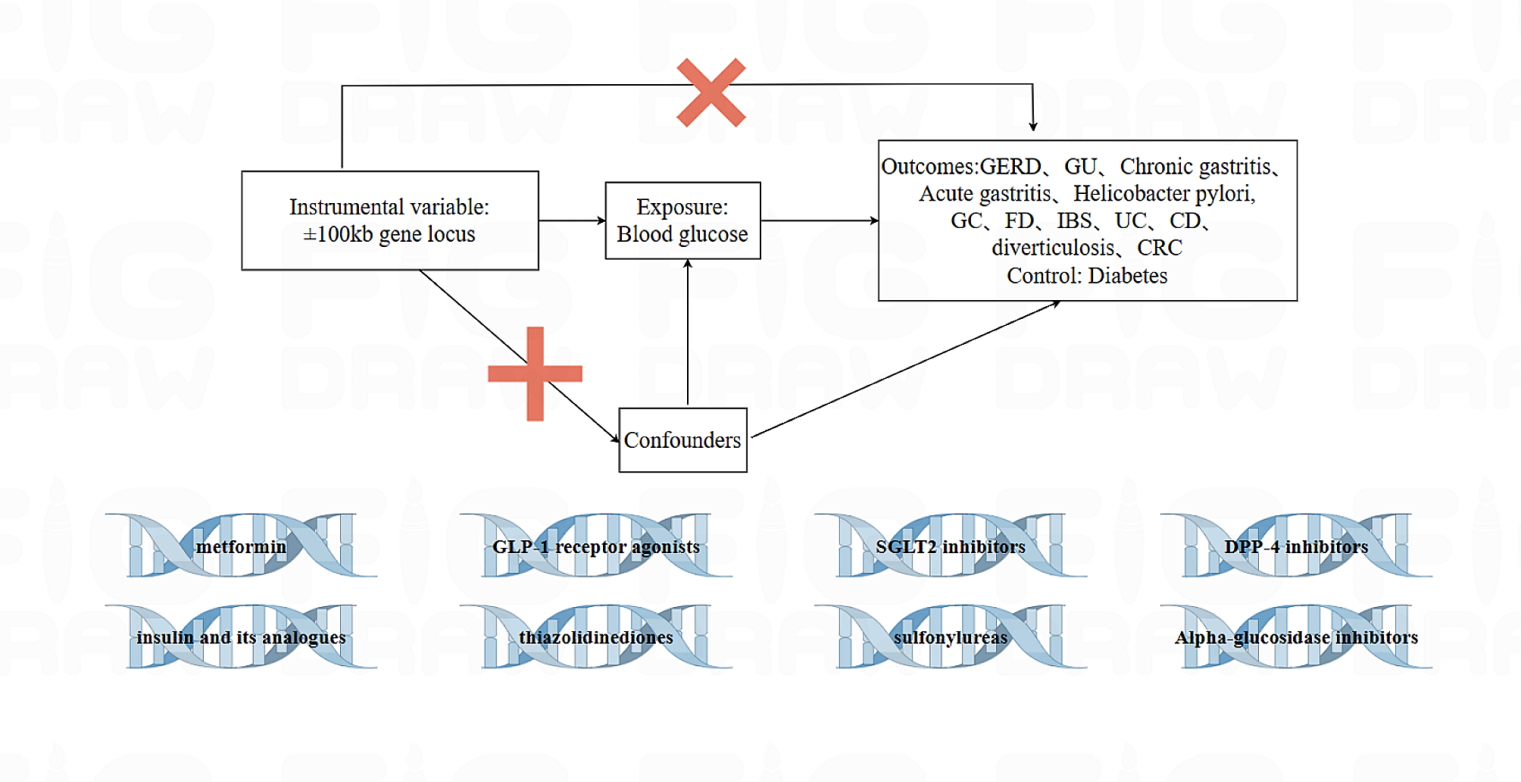
This is a flowchart of the study design and the MR Analysis process The causal relationship between antidiabetic drugs and gastrointestinal disorders was assessed by a two-sample MR analysis dealing with exposure and outcome data. Three core assumptions were met: (1) IVs and exposure (antidiabetic drugs) are strongly correlated; (2) there is no correlation between IVs and confounders; and (3) there is no direct correlation between IVs and outcomes, and their effect on outcomes can only be reflected by the degree of exposure

We estimated the causal relationship between antidiabetic drugs and gastrointestinal diseases by a TSMR study (Fig. 1). We selected the major antidiabetic drugs, including metformin, GLP-1 receptor agonists, SGLT2 inhibitors, DPP-4 inhibitors, insulin and its analogs, thiazolidinediones, sulfonylureas, and alpha-glucosidase inhibitors [28, 29]. Genetic variants associated with blood glucose levels in these drug-target genes were identified to proxy the drug-target effect [27]. We then analyzed the effect of these genetic variants on gastrointestinal disorders using MR.

### Data source

Aggregate data for all genome-wide association studies (GWAS) used in the study were obtained from the IEU Open GWAS database (https://gwas.mrcieu.ac.uk/datasets) [30] and are detailed in Table 1.

**Table 1:** Summary of the GWAS included in this MR study

Trait	Dataset	Sample size	Number of SNPs	Population
**Exposure**				
Blood glucose	ebi-a-GCST90025986	400,458	4,218,897	European
**Outcome**				
GERD	ebi-a-GCST90000514	602,604	2,320,781	European
gastric ulcer	ebi-a-GCST90018851	474,278	24,178,780	European
Chronic gastritis	ukb-b-6716	463,010	9,851,867	European
Acute gastritis	finn-b-K11_ACUTGASTR	NA	16,380,389	European
Helicobacter pylori	ukb-b-531	462,933	9,851,867	European
GC	ebi-a-GCST90018849	476,116	24,188,662	European
FD	finn-b-K11_FUNCDYSP	189,695	16,380,380	European
IBS	ukb-b-2592	462,933	9,851,867	European
UC	ukb-b-7584	462,933	9,851,867	European
CD	ukb-a-552	337,199	10,894,596	European
Diverticulosis	finn-b-K11_DIVERTIC	182,423	16,380,412	European
CRC	ieu-b-4965	377,673	11,738,639	European
**Positive control outcomes**				
Diabetes	ukb-b-12,948	462,933	9,851,867	European

GERD: Gastroesophageal reflux disease; GU: Stomach ulcers; GC: Gastric cancer; FD: functional dyspepsia; IBS: Irritable bowel syndrome; UC: Ulcerative colitis; CD: Crohn’s Disease; CRC: Colorectal cancer; MR: Mendelian randomization; GWAS: Genome-wide association studies; SNPs: Single nucleotide polymorphisms

### Genetic instrumentation for antidiabetic drugs

We used the DrugBank (go.drugbank.com) and ChEMBL (ebi.ac.uk/chembl) databases to identify genes encoding the target proteins of these antidiabetic drugs [31, 32] (Table 2). Based on previous studies [33], we selected genome-wide salient variants (*p* < 5 × 10^− 8^) associated with blood glucose levels and SNPs within a 100 kb window of the target gene for each drug to determine exposure to antidiabetic drugs [34]. The instrumental variant SNPs were located within ± 100 kb of the antidiabetic drug site, ensuring that the linkage imbalance was not too strong (r^2^ < 0.3). We estimated the F-statistic of the instrument, retaining only SNPs with an F > 10 to avoid weak instrument bias [35]Since antidiabetic drugs are used to treat diabetes, we used diabetes as a positive control [36], utilizing GWAS pooled data that included 462,933 samples.

**Table 2:** Target genes of antidiabetic drugs from DrugBank and ChEMBL databases

Drug class	Encoding genes of target proteins		Gene location
	DrugBank	ChEMBL	
Metformin	PRKAB1	Fifty-eight encoding genes	(NA)
	ETFDH	GPD2	
GLP-1 receptor agonists	GLP1R	GLP1R	Chr6: 39,016,557 − 39,059,079
SGLT2 inhibitors	SLC5A2	SLC5A2	Chr16: 31,494,323 − 31,502,181
DDP-4 inhibitors	DPP4	DPP4	Chr2: 162,848,755 −162,930,904
Insulin and its analogues	INSR	INSR	Chr19: 7,112,266-7,294,425
Thiazolidinediones	PPARG	PPARG	Chr3: 12,328,867 − 12,475,855
Sulfonylureas	KCNJ11	KCNJ11	Chr11: 17,386,719 − 17,410,878
	ABCC8	ABCC8	Chr11: 17,414,045 − 17,498,441
Alpha-glucosidase inhibitors	M6PR	M6PR	Chr17:78,075,380− 78,093,680

GLP-1: glucagon-like peptide-1; SGLT2: sodium-glucose cotransporter-2; DPP4: dipeptidyl peptidase-4; NA: not applicable

### Statistical analysis

We used MR analysis to align drug-targeted instrumental variables with outcome datasets. The inverse variance weighting (IVW) method was employed as the primary analytical method, disregarding the intercept term and using the reciprocal of the outcome variance (se squared) as a fitting weight [37]. The weighted median, MR-Egger regression, simple mode, and weighted mode were used as supplementary analysis methods to further improve the credibility and accuracy of the results [38]. To avoid heterogeneity in instrumental variables (IVs), we used Cochran’s Q test, where *p* > 0.05 indicated no significant heterogeneity [39]. The IVW method requires careful consideration of IVs to ensure their non-pleiotropic nature, as biased results may arise otherwise. Pleiotropy was assessed using MR-Egger regression to ensure that IVs did not introduce bias through alternative pathways. MR-Egger regression incorporates an intercept term and uses the inverse of the outcome variance (se squared) as a weighting factor for fitting. If the MR-Egger intercept is close to 0 or *p* > 0.05, it indicates no evidence of pleiotropic effects in IVs [40]. The “leave-one-out” method was used to systematically eliminate each SNP, calculate the meta-effect of the remaining SNPs, and assess whether there were any alterations in the results upon removal of each individual SNP. This approach aimed to mitigate potential influences from specific SNPs on our findings [41].

All analyses were performed using the “Two Sample MR” package [42] in R version 4.3.1. The threshold of statistical significance was set at *p* < 0.05.

## Results

### Selection and validation of genetic instruments

A total of 400,458 samples were included in the aggregated data of blood glucose GWAS. Through a rigorous selection process, no suitable genetic instruments were found for drugs such as metformin, SGLT2 inhibitors, DDP-4 inhibitors, Insulin and its analogs, Thiazolidinediones, Alpha-glucosidase inhibitors, etc. However, two SNPs were identified for GLP-1 receptor agonists, with one having an F-value of 11.5 after excluding those with F < 10. For sulfonylureas, three SNPs were identified with F-values of 11.4, 14.4, and 36.6, respectively. The F-values of these SNPs are all above the threshold of 10, indicating that our study largely avoids weak instrument bias.

### Positive control analysis

**Fig. 2:**
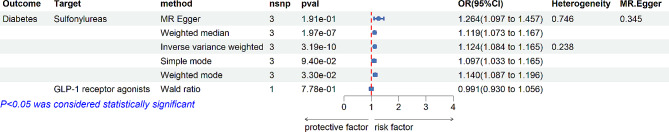
Relationship between GLP-1 receptor agonists, sulfonylureas and diabetes mellitus nsnp (number of single nucleotide polymorphisms), OR (odds ratio), CI (confidence interval)

The pharmacogenetic analysis of antidiabetic drugs and diabetes mellitus showed positive results for sulfonylureas (IVW: OR [95% CI] = 1.12 [1.07–1.17], *p* = 1.97E-07). In contrast, the analysis for GLP-1 receptor agonists was negative (IVW: OR [95% CI] = 0.99 [0.93–1.06], *p* = 0.78) (Fig. 2). These positive control analyses validated the genetic instruments for sulfonylureas but not for GLP-1 receptor agonists.

### MR analysis of drug targets in gastrointestinal diseases

**Fig. 3:**
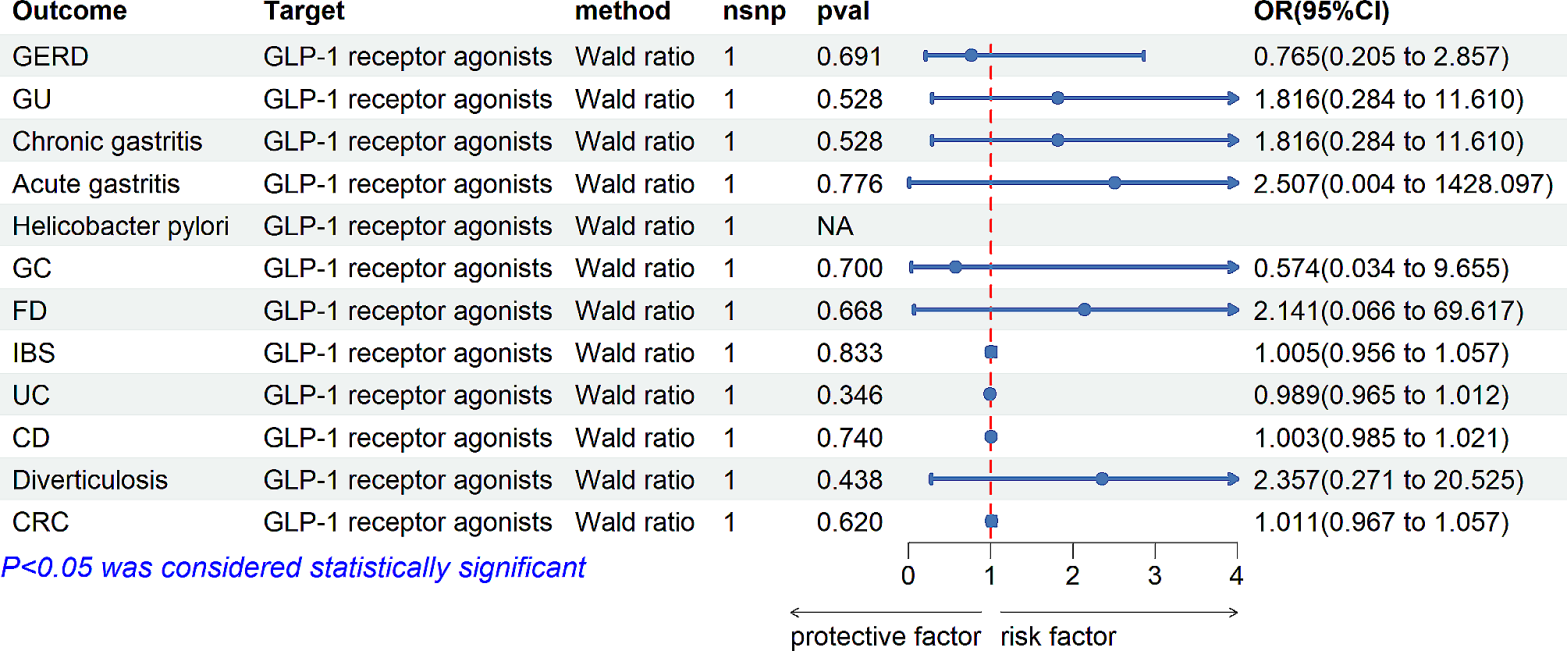
The relationship between GLP-1 receptor agonists and gastrointestinal disorders nsnp (number of single nucleotide polymorphisms), OR (odds ratio), CI (confidence interval), GERD: Gastroesophageal reflux disease;; GC: Gastric cancer; functional dyspepsia (FD); IBS: Irritable bowel syndrome; UC: Ulcerative colitis; CD: Crohn’s Disease; CRC: Colorectal cancer

**Fig. 4:**
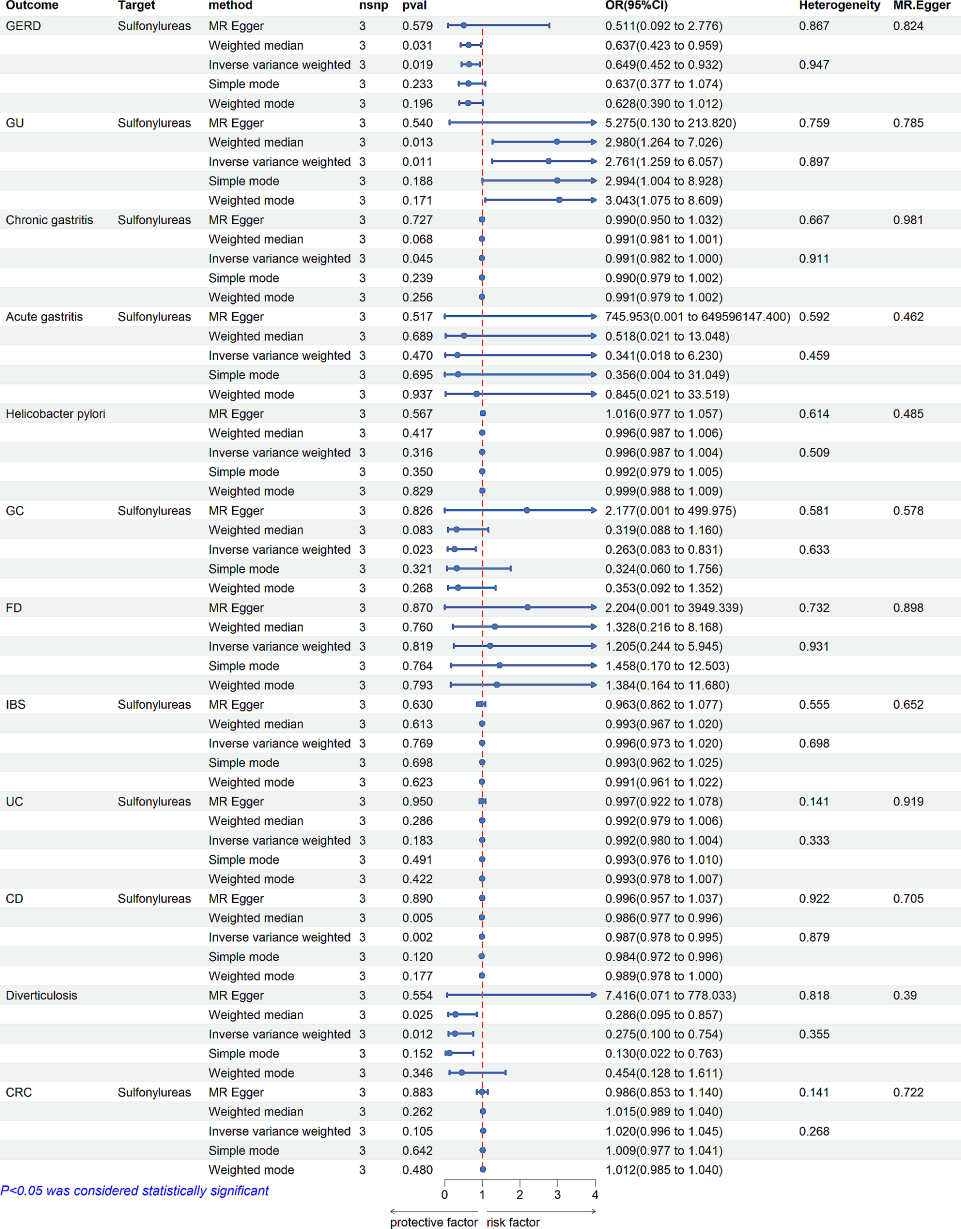
The relationship between Sulfonylureas and gastrointestinal disorders nsnp (number of single nucleotide polymorphisms), OR (odds ratio), CI (confidence interval), GERD: Gastroesophageal reflux disease; GC: Gastric cancer; functional dyspepsia (FD); IBS: Irritable bowel syndrome; UC: Ulcerative colitis; CD: Crohn’s Disease; CRC: Colorectal cancer

Drug-target MR Analysis was conducted to explore the causal relationship between antidiabetic drugs and gastrointestinal disorders. Genetic proxies for GLP-1 receptor agonists did not show a causal association with gastrointestinal disorders (Fig. 3). However, sulfonylureas were found to reduce the risk of CD (IVW: OR [95%CI] = 0.986 [0.978, 0.995], *p* = 1.99 × 10^− 3^; Weighted median: OR [95%CI] = 0.986 [0.977, 0.996], *p* = 5.30 × 10^− 3^), GERD (IVW: OR [95%CI] = 0.649 [0.452, 0.932], *p* = 1.90 × 10^− 2^; Weighted median: OR [95%CI] = 0.472 [0.242, 0.922], *p* = 2.78 × 10^− 2^), and chronic gastritis (IVW: OR[95%CI] = 0.991 [0.982, 0.999], *p* = 4.50 × 10^− 2^). Conversely, sulfonylureas increased the incidence of GU in patients with diabetes (IVW: OR [95%CI] = 2.761 [1.259, 6.057], *p* = 1,12 × 10 ^− 2^; Weighted median: OR [95%CI] = 2.980 [1.264, 7.026], *p* = 1.26 × 10^− 2^) (Fig. 4).

### Sensitivity analysis

Cochran’s Q-test showed no evidence of heterogeneity (*p* > 0.05). The MR-Egger intercept analysis indicated no horizontal pleiotropy (*p* > 0.05). The robustness of our conclusions was further supported by the leave-one-out sensitivity (Fig. 4). Thus, our MR analysis proved to be reliable and robust.

## Discussion

We conducted large-scale MR analyses on gastrointestinal diseases using data from the IEU Open GWAS database. Our study investigated the causal relationship of seven common antidiabetic drug targets–metformin, GLP-1 receptor agonists, SGLT2 inhibitors, DPP-4 inhibitors, insulin and its analogs, thiazolidinediones, sulfonylureas and alpha-glucosidase inhibitors–on various gastrointestinal diseases. These diseases included GERD, GU, chronic gastritis, acute gastritis, Helicobacter pylori infection, gastric cancer, FD, IBS, UC, CD, diverticulosis, and CRC. Our MR results showed that SGLT2 inhibitors, DDP-4 inhibitors, Insulin and its analogs, Thiazolidinedione, and other drugs did not have significant effects on gastrointestinal diseases and were not further analyzed. GLP-1 receptor agonists did not affect gastrointestinal tract diseases. Notably, sulfonylureas were found to reduce the risk of CD, GERD, and chronic gastritis but increase the risk of developing stomach ulcers.

The mechanisms underlying the preventive effects of sulfonylureas on the development of CD, GERD, chronic gastritis, and increased risk of GU remain unexplored. However, examining the pathways through which diabetes causes gastrointestinal diseases and reviewing clinical studies on sulfonylureas provide some insights.

Previous retrospective studies have indicated that gastrointestinal disorders are common complications of diabetes [43, 44]. MR studies have demonstrated an elevated risk of GERD [45] and gastritis [13] in individuals with diabetes. An MR study conducted by Xiang Xiao et al. revealed that type 2 diabetes reduces the risk of inflammatory bowel disease [46]. The pathogenesis of gastrointestinal complications in diabetes has been extensively explored in numerous articles.

Studies have shown that people with diabetes are more likely to develop GERD [45]. Animal studies have identified glucose-responsive neurons in the central nervous system, suggesting that high blood glucose may alter vagal efferent activity [47]. Clinical studies have shown that diabetes mellitus leads to dysfunction of the parasympathetic component of the autonomic nervous system, resulting in esophageal innervation dysfunction and gastroesophageal reflux disease [48, 49]. Sulfonylureas can reduce the incidence of GERD by modulating Drp-1-mediated oxidative stress and apoptosis, which ameliorates peripheral neuropathy [50, 51]. Crohn’s disease is a recurrent systemic inflammatory disease primarily involving the gastrointestinal tract, with extraintestinal manifestations and associated immune disorders [52, 53]. Studies have shown that mast cells release biologically active mediators such as serine proteases mMCP-6 and Prss31, which are involved in the development of acute colitis [54]. Animal experiments by Vijay Chidrawar et al. have shown that sulfonylureas can ameliorate inflammation by blocking cystic fibrosis transmembrane conductance modulator channels on mast cells [55]. Chronic gastritis is an inflammatory disease of the gastric mucosa [56]. P Kashyap et al. demonstrated that oxidative stress plays a crucial role in diabetes mellitus, triggering gastrointestinal complications [39]. Oxidative stress results from an imbalance between reactive oxygen species (ROS) production and endogenous antioxidant defense mechanisms [40]. Experiments in diabetic rats showed that sulfonylureas have significant antioxidant effects and can be used to treat Crohn’s disease and chronic gastritis by attenuating oxidative stress-induced damage. In addition, abnormal NLRP3 inflammasome activity has been identified as a key factor in the pathogenesis of Crohn’s disease and chronic gastritis [57, 58]. Inhibition of NLRP3 inflammasome by sulfonylureas can effectively inhibit the release of major proinflammatory cytokines/chemokines, which can effectively treat Crohn’s disease and chronic gastritis [58, 59].

The main factors leading to gastric ulcers include the presence of strong acids and high levels of proteolytic activity (pepsin) in gastric secretions [60]. Control tests by H.A.F Ismail et al. demonstrated that nicorandil could provide gastric protection by opening K (ATP) channels, scavenging free radicals, reducing pepsin and gastric acid secretion, and preventing harmful elevation of nitric oxide during water immersion-restraint stress [61]. However, sulfonylureas reduce blood glucose by inhibiting potassium flux in the ATP-dependent potassium channel (KATP) and inducing glucose-independent insulin release from β-cells [62]. As K (ATP) channel blockers [63], sulfonylureas can negate the protective effects of opening these channels, thereby increasing the risk of stomach ulcers.

Furthermore, a comparative study showed that hypoglycemia is associated with increased total pepsin secretion [64]. Sulfonylureas stimulate insulin release from pancreatic cells and have an extrapancreatic hypoglycemic effect, making them more likely to induce hypoglycemia [65]. The most common side effect of sulfonylureas is hypoglycemia [66]. Hence, one of the mechanisms by which sulfonylureas increase the risk of gastric ulcer may be attributed to their side effect of causing hypoglycemia, which subsequently leads to increased pepsin secretion and eventually gastric ulcer.

We employed MR studies to establish causal associations between sulfonylureas and CD, GERD, chronic gastritis, and GU. (1) Our MR study utilized genetic variants as proxies for antidiabetic drugs to mitigate confounding factors and reverse causality issues that may have affected previous studies. (2) Our analysis focused on individuals of European ancestry for both exposure and individual outcome data in order to effectively minimize potential association effects arising from population stratification. (3) In our study design, we selected genetic variants within a 100 kb window of the coding gene using a threshold of 5 × 10^− 8^ as instrumental variables. Additionally, we filtered out instrumental variables with an F value of less than 10 to improve the reliability of our results. (4) To ensure the robustness of our findings, we conducted positive control analysis, heterogeneity tests, pleiotropy tests, and sensitivity analyses throughout our study process. These measures further enhance the reliability of our results.

However, it is important to acknowledge certain limitations in our study. Firstly, the TSMR analysis was solely based on individuals of European ancestry, which restricts generalizability beyond this specific population group. Caution should be exercised when extrapolating these findings to racially and ethnically diverse populations. Secondly, drug target MR can assess long-term drug effects but cannot replace clinical trials for verifying short-term drug effects. MR provides a way to analyze the causal relationship between exposure and outcome but cannot replace clinical trials. Also, our study did not investigate the association between other antidiabetic drugs and gastrointestinal diseases. Lastly, we used GWAS summary data from the IEU Open GWAS database. The data sources were not stratified, so further stratified analysis could not be performed.

## Conclusions

This study provides evidence that sulfonylureas may prevent CD, GERD, and chronic gastritis while increasing the risk of GU. These findings could help diabetic patients in managing and preventing certain gastrointestinal diseases. Further clinical trials are necessary to elucidate the potential mechanistic pathways between sulfonylureas and these conditions. Additionally, promoting better use of antidiabetic drugs is essential.

### Electronic supplementary material

Below is the link to the electronic supplementary material.

Supplementary Material 1: Leave one method for analysis chart. **A**: Exposure: Sulfonylureas, outcome: GERD; **B**: Exposure: Sulfonylureas, outcome: GU; **C**: Exposure: Sulfonylureas, outcome: Chronic gastritis; **D**: Exposure: Sulfonylureas, outcome: Acute gastritis; **E**: Exposure: Sulfonylureas, outcome: GC; **F**: Exposure: Sulfonylureas, outcome: IBS; **G**: Exposure: Sulfonylureas, outcome: UC; **H**: Exposure: Sulfonylureas, outcome: CD; **I**: Exposure: Sulfonylureas, outcome: CRC; **J**: Exposure: Sulfonylureas, outcome: Diabetes; **K**: Exposure: Sulfonylureas, outcome: Helicobacter pylori; **L**: Exposure: Sulfonylureas, outcome: FD); **M**: Exposure: Sulfonylureas, outcome: diverticulosis

## Data Availability

No datasets were generated or analysed during the current study.

## Abbreviations

IVs: Instrumental variables
TSMR: Two-sample Mendelian randomization
GWAS: Genome-wide association study
SNPs: Single nucleotide polymorphisms
IVW: Inverse variance weighted
GERD: Gastroesophageal reflux disease
GU: Gastric ulcer
GC: Gastric cancer
FD: Functional dyspepsia
IBS: Irritable bowel syndrome
UC: Ulcerative colitis
CD: Crohn’s disease
CRC: Colorectal cancer
ROS: Reactive oxygen species
