# Supplementary material for: The causal relationship between anti-diabetic drugs and gastrointestinal disorders: a drug-targeted mendelian randomization study
Source: Diabetol Metab Syndr. 2024 Jun 26;16:141. doi: 10.1186/s13098-024-01359-z (PMC11201305; doi:10.1186/s13098-024-01359-z)
Supplement: Supplementary file 1 — Supplementary Material 1: Leave one method for analysis chart. A: Exposure: Sulfonylureas, outcome: GERD; B: Exposure: Sulfonylureas, outcome: GU; C: Exposure: Sulfonylureas, outcome: Chronic gastritis; D: Exposure: Sulfonylureas, outcome: Acute gastritis; E: Exposure: Sulfonylureas, outcome: GC; F: Exposure: Sulfonylureas, outcome: IBS; G: Exposure: Sulfonylureas, outcome: UC; H: Exposure: Sulfonylureas, outcome: CD; I: Exposure: Sulfonylureas, outcome: CRC; J: Exposure: Sulfonylureas, outcome: Diabetes; K: Exposure: Sulfonylureas, outcome: Helicobacter pylori; L: Exposure: Sulfonylureas, outcome: FD); M: Exposure: Sulfonylureas, outcome: diverticulosis [file 13098_2024_1359_MOESM1_ESM.pdf]

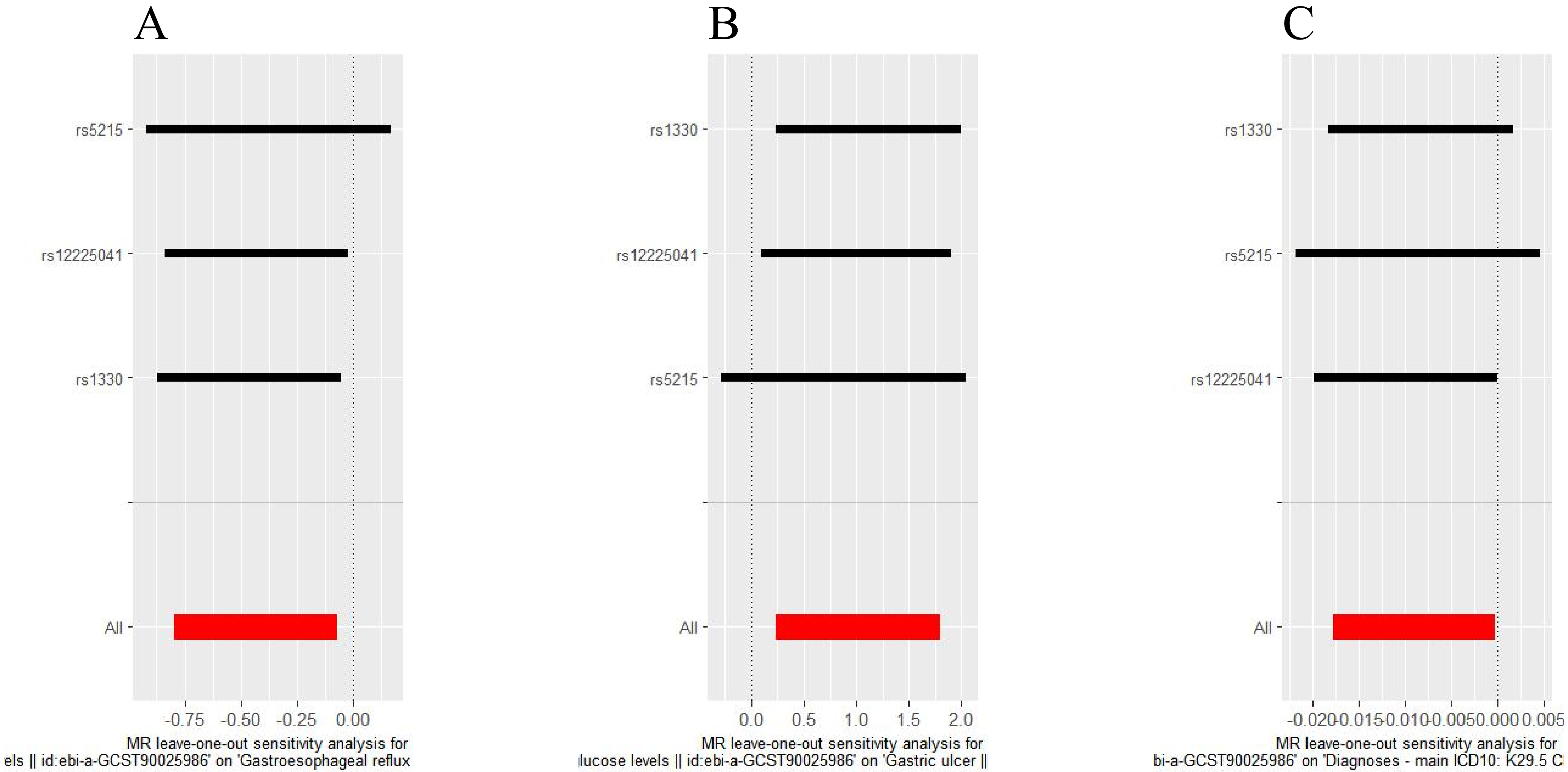

A
rs5215
rs12225041
rs1330
All
-0.75 -0.50 -0.25 0.00
MR leave-one-out sensitivity analysis for
els || id:ebi-a-GCST90025986' on 'Gastroesophageal reflux
B
rs1330
rs12225041
rs5215
All
0.0 0.5 1.0 1.5 2.0
MR leave-one-out sensitivity analysis for
lucose levels || id:ebi-a-GCST90025986' on 'Gastric ulcer ||
C
rs1330
rs5215
rs12225041
All
-0.020 -0.015 -0.010 -0.005 0.000 0.005
MR leave-one-out sensitivity analysis for
bi-a-GCST90025986' on 'Diagnoses - main ICD10: K29.5 C

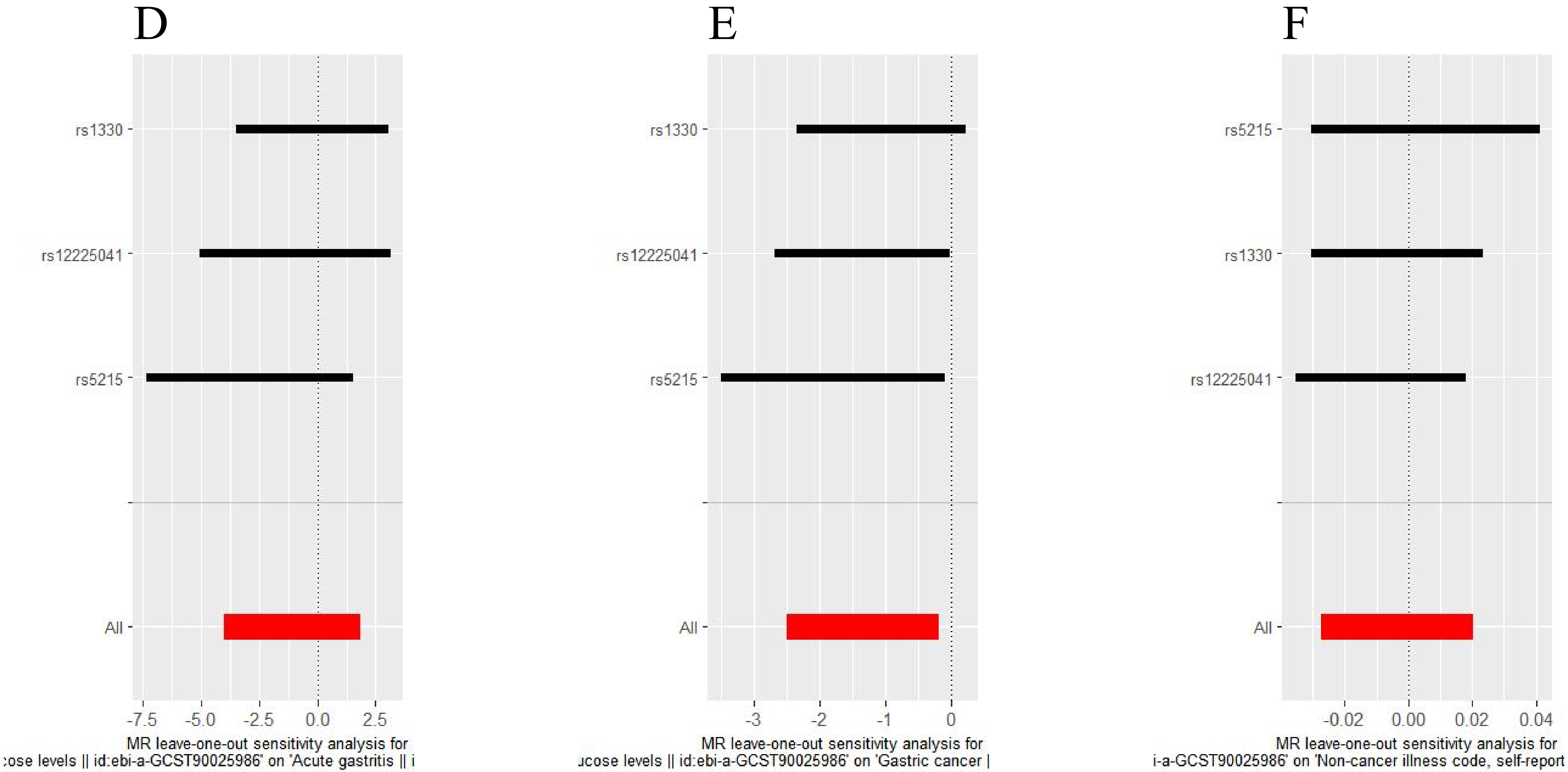
D
rs1330
rs12225041
rs5215
All
-7.5
-5.0
-2.5
0.0
2.5
MR leave-one-out sensitivity analysis for
:ose levels || id:ebi-a-GCST90025986' on 'Acute gastritis || i
E
rs1330
rs12225041
rs5215
All
-3
-2
-1
0
MR leave-one-out sensitivity analysis for
ucose levels || id:ebi-a-GCST90025986' on 'Gastric cancer |
F
rs5215
rs1330
rs12225041
All
-0.02
0.00
0.02
0.04
MR leave-one-out sensitivity analysis for
i-a-GCST90025986' on 'Non-cancer illness code, self-report

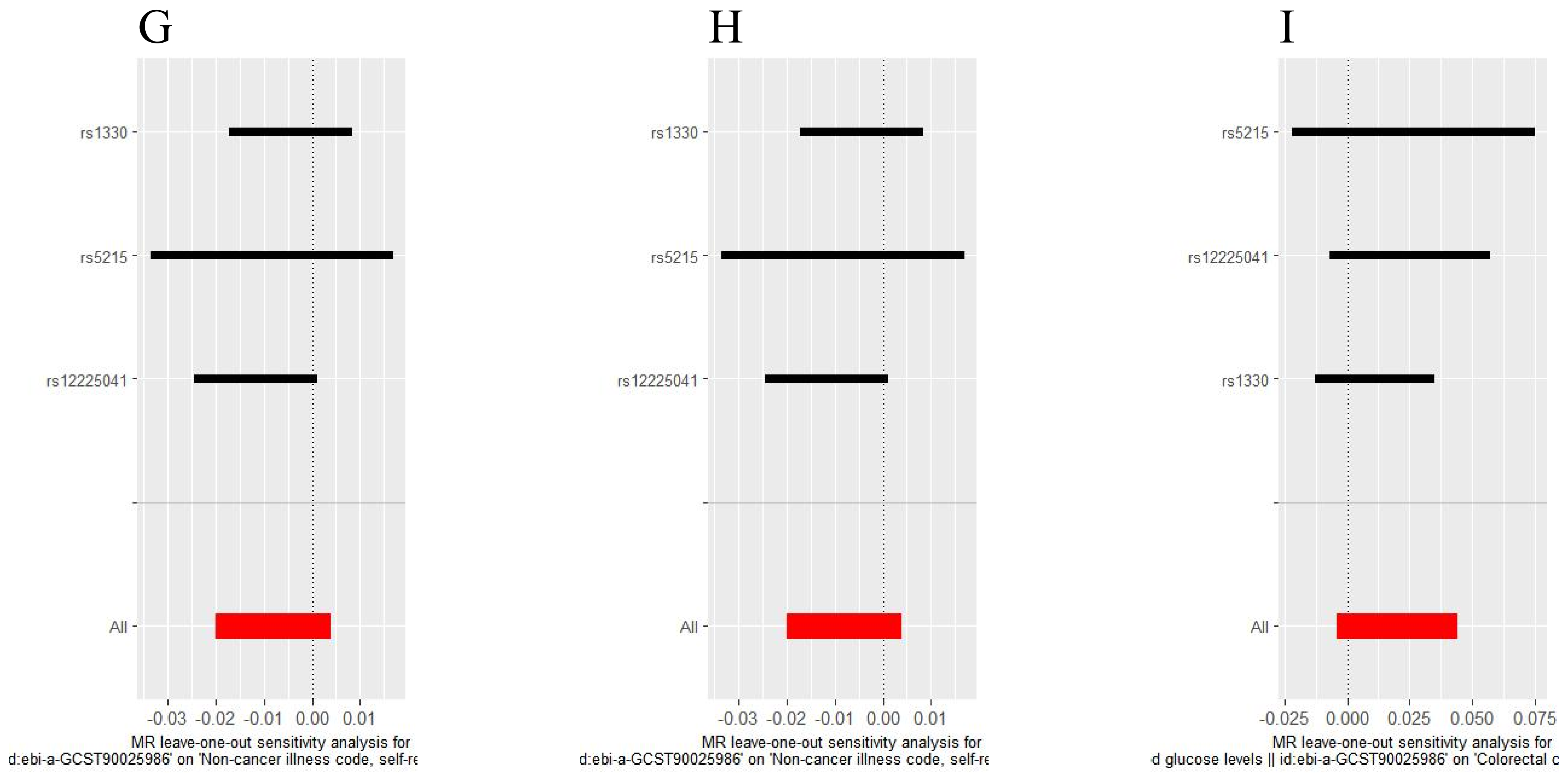
G
rs1330
rs5215
rs12225041
All
-0.03 -0.02 -0.01 0.00 0.01
MR leave-one-out sensitivity analysis for
d:ebi-a-GCST90025986' on 'Non-cancer illness code, self-re
H
rs1330
rs5215
rs12225041
All
-0.03 -0.02 -0.01 0.00 0.01
MR leave-one-out sensitivity analysis for
d:ebi-a-GCST90025986' on 'Non-cancer illness code, self-re
I
rs5215
rs12225041
rs1330
All
-0.025 0.000 0.025 0.050 0.075
MR leave-one-out sensitivity analysis for
d glucose levels || id:ebi-a-GCST90025986' on 'Colorectal c

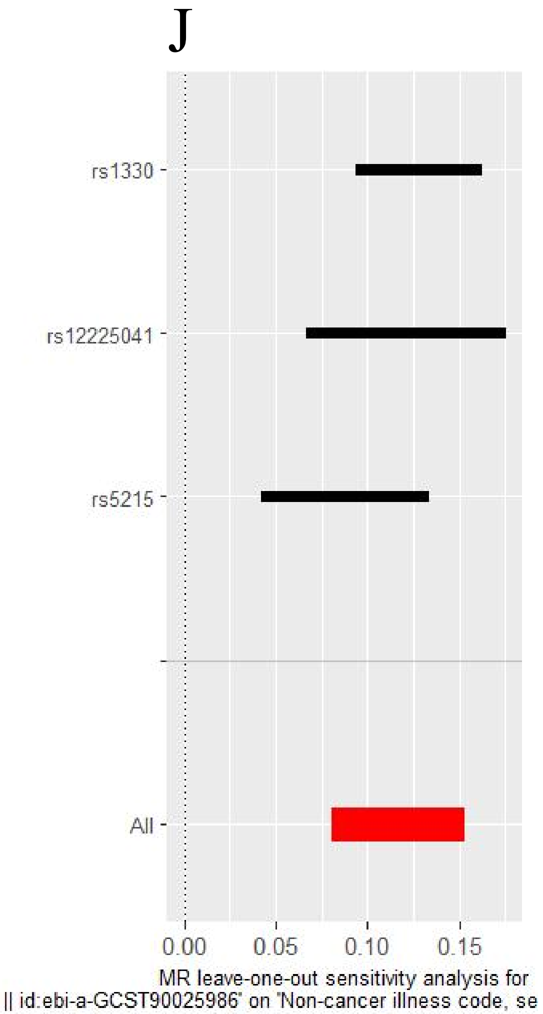
J
rs1330
rs12225041
rs5215
All
0.00
0.05
0.10
0.15
MR leave-one-out sensitivity analysis for
|| id:ebi-a-GCST90025986' on 'Non-cancer illness code, se

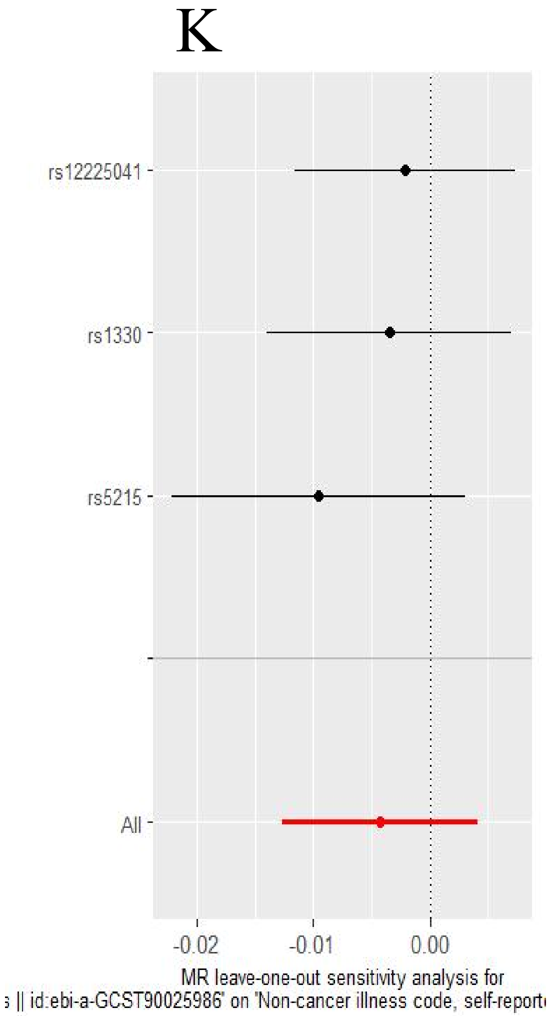
K
rs12225041
rs1330
rs5215
All
-0.02
-0.01
0.00
MR leave-one-out sensitivity analysis for
|| id:ebi-a-GCST90025986' on 'Non-cancer illness code, self-report

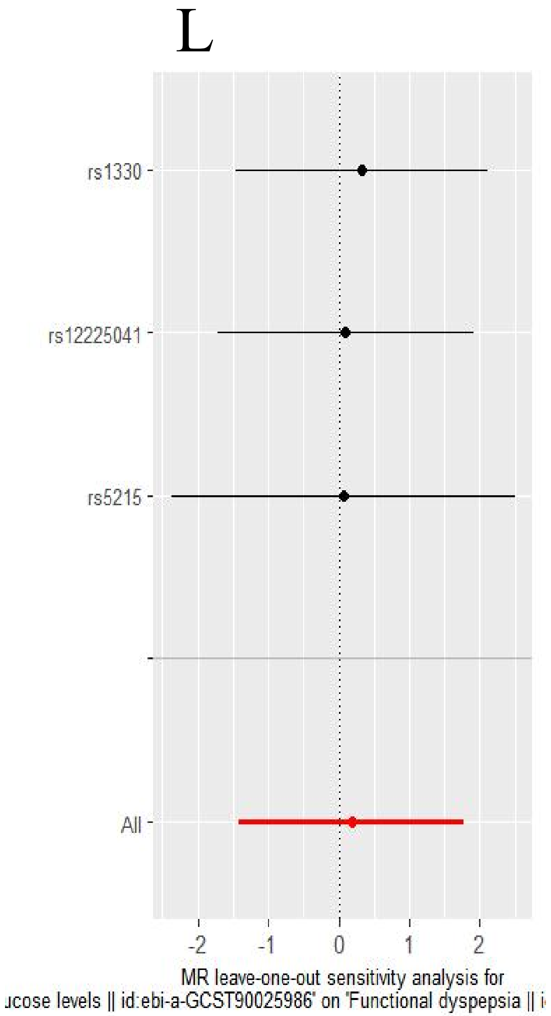
L
rs1330
rs12225041
rs5215
All
-2
-1
0
1
2
MR leave-one-out sensitivity analysis for
ucose levels || id:ebi-a-GCST90025986' on 'Functional dyspepsia ||

M

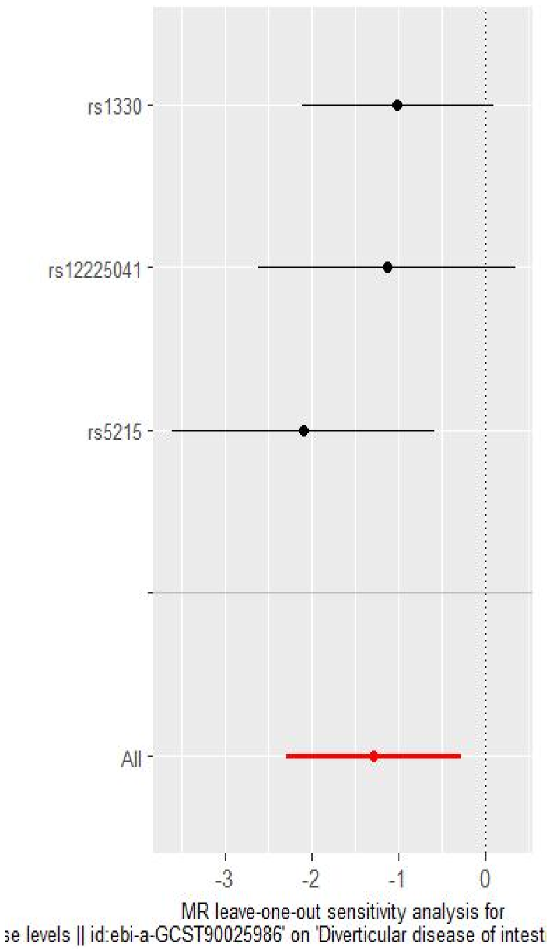
rs1330
rs12225041
rs5215
All
-3
-2
-1
0
MR leave-one-out sensitivity analysis for
se levels || id:ebi-a-GCST90025986' on 'Diverticular disease of intest
